# Supplementary material for: Prognosis and immune landscape of bladder cancer can be predicted using a novel miRNA signature associated with cuproptosis
Source: PeerJ. 2024 Nov 29;12:e18530. doi: 10.7717/peerj.18530 (PMC11610463; doi:10.7717/peerj.18530)
Supplement: Supplemental Information 1 [file peerj-12-18530-s001.docx]

**Table S1. Name of primer**

| Name of primer Sequence of primer | |
| --- | --- |
| hsa-miR-125b-2-3p  hsa-miR-145-3p  hsa-miR-409-3p  hsa-miR-625-3p  U6 | 5ʹ-CAAGTCAGGCTCTTGGGACAA- 3ʹ  5ʹ-GGATTCCTGGAAATACTGTTCTA- 3ʹ  5ʹ-ATGTTGCTCGGTGAACCCCTA- 3ʹ  5ʹ-GACTATAGAACTTTCCCCCTCA- 3ʹ  Forward: 5′-CTCGCTTCGGCAGCACATATACT-3′  Reverse: 5′-ACGCTTCACGAATTTGCGTGTC-3′ |

| **Table S2. Probes for FISH** | | | |
| --- | --- | --- | --- |
| Name of miRNA | FISH Probe | Lable | Source |
| hsa-miR-125b-2-3p | GTCCCAAGAGCC+TGACT+TGTGA | Cy3 | Genepharma |
| hsa-miR-145-3p | AGAACAGTAT+TTCCAGGAA+TCC | Cy3 | Genepharma |
| hsa-miR-409-3p | AGGGGT+TCACCGAGCAACAT+TC | Cy3 | Genepharma |
| hsa-miR-625-3p | TGAGGGGGAAAGT+TCTA+TAGTC | Cy3 | Genepharma |

| **Table S3. Sequence of mimics** | | |
| --- | --- | --- |
| Mimics | Sequence | Source |
| hsa-miR-125b-2-3p | UCACAAGUCAGGCUCUUGGGAC | Genepharma |
|  | CCCAAGAGCCUGACUUGUGAUU | Genepharma |
| hsa-miR-145-3p | GGAUUCCUGGAAAUACUGUUCU | Genepharma |
|  | AACAGUAUUUCCAGGAAUCCUU | Genepharma |
| hsa-miR-409-3p | GAAUGUUGCUCGGUGAACCCCU | Genepharma |
|  | GGGUUCACCGAGCAACAUUCUU | Genepharma |
| hsa-miR-625-3p | GACUAUAGAACUUUCCCCCUCA | Genepharma |
|  | AGGGGGAAAGUUCUAUAGUCUU | Genepharma |

| **Table 4. Sequence of inhibor** | | |
| --- | --- | --- |
| Inhibitor | Sense and antisence | Source |
| hsa-miR-125b-2-3p | 5'-GUCCCAAGAGCCUGACUUGUGA-3' | Genepharma |
|  | 3'-CAGGGUUCUCGGACUGAACACU-5' | Genepharma |
| hsa-miR-145-3p | 5'-AGAACAGUAUUUCCAGGAAUCC-3' | Genepharma |
|  | 3'-UCUUGUCAUAAAGGUCCUUAGG-5' | Genepharma |
| hsa-miR-409-3p | 5'-AGGGGUUCACCGAGCAACAUUC-3' | Genepharma |
|  | 3'-UCCCCAAGUGGCUCGUUGUAAG-5' | Genepharma |
| hsa-miR-625-3p | 5'-UGAGGGGGAAAGUUCUAUAGUC-3' | Genepharma |
|  | 3'-ACUCCCCCUUUCAAGAUAUCAG-5' | Genepharma |
