# Supplementary material for: Prognosis and immune landscape of bladder cancer can be predicted using a novel miRNA signature associated with cuproptosis
Source: PeerJ. 2024 Nov 29;12:e18530. doi: 10.7717/peerj.18530 (PMC11610463; doi:10.7717/peerj.18530)
Supplement: Supplemental Information 2 [file peerj-12-18530-s002.docx]

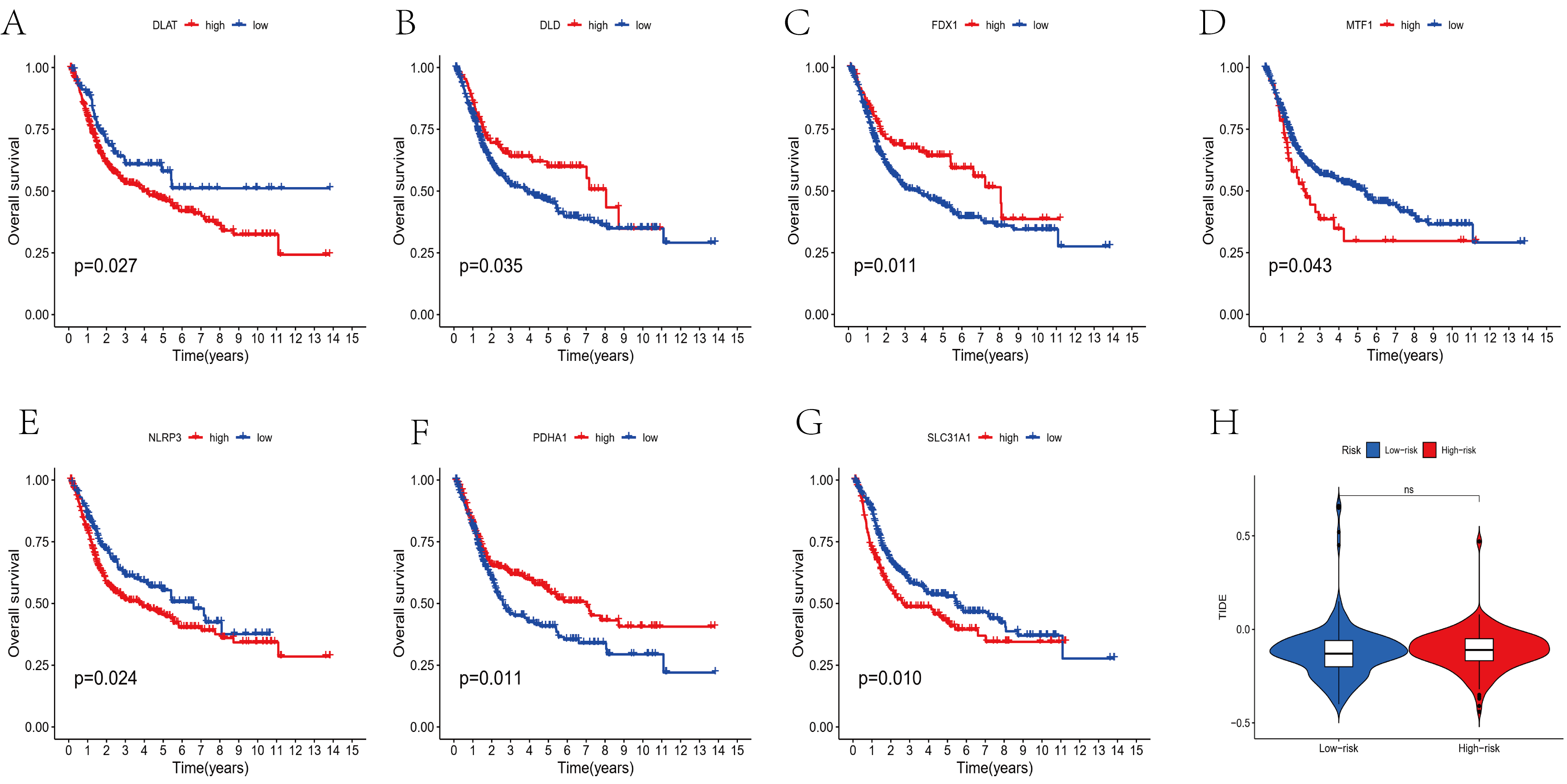


**Figure S1.** (**A-G**) prognostic value analysis of Cuproptosis related genes, DLAT, DLD, FDX1, MTF1, NLRP3, PDHA1, SLC31A1. (H) The score of TIDE between the low and high risk groups.


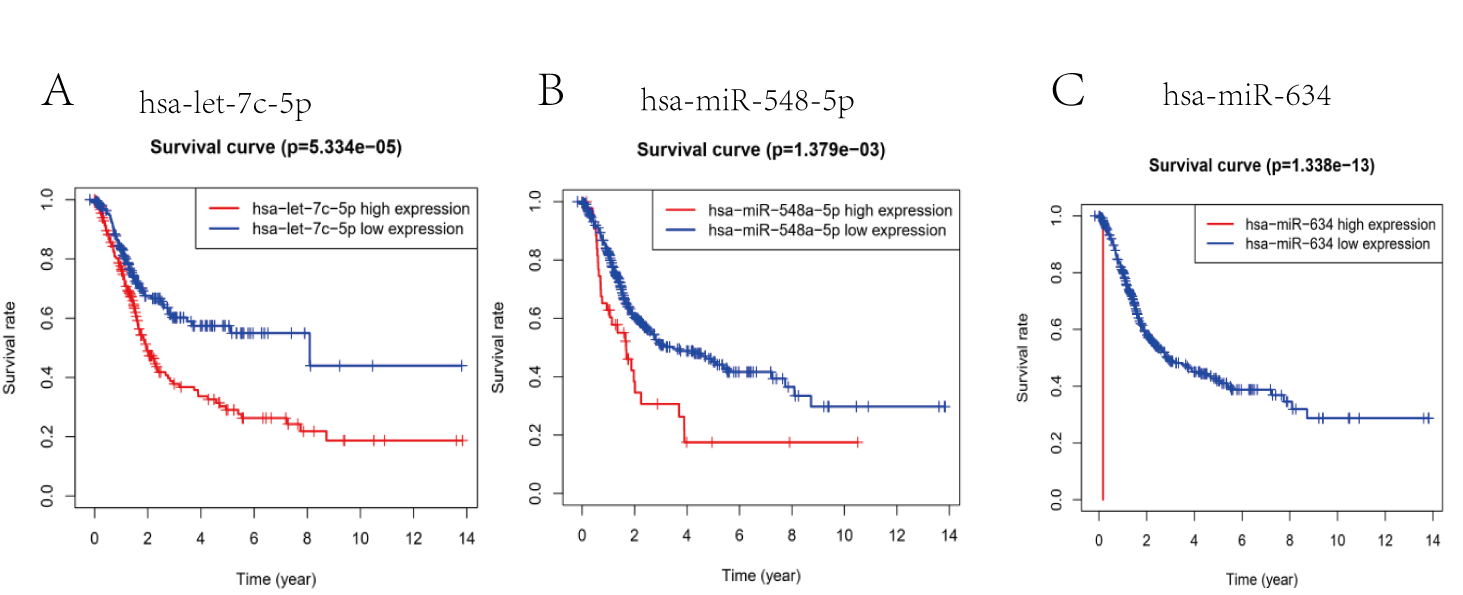
**Figure S2.** (**A-C**) prognostic value analysis of hsa-let-7c-5p, hsa-miR-548-5p, hsa-miR-634.
